# The Family Health Strategy Influence on the Human Papillomavirus Vaccine Acceptance in a Peripheral Community of the Brazilian Amazon Region

**DOI:** 10.1089/heq.2022.0009

**Published:** 2022-11-18

**Authors:** Glenda Roberta Oliveira Naiff Ferreira, Julie Ane da Silva Formigosa, Ana Luisa Brandão de Carvalho Lira, Renata Karina Reis, Elucir Gir, Wanne Letícia Santos Freitas, Jacira Nunes Carvalho, Lucia Hisako Takase Gonçalves, Eliã Pinheiro Botelho, Aline Maria Pereira Cruz Ramos

**Affiliations:** ^1^ Graduate Nursing Program at the Federal University of Pará (UFPA), Belém, Pará, Brazil.; ^2^ Graduate Nursing Program at the Federal University of Rio Grande do Norte (UFRGN), Natal, Rio Grande do Norte, Brazil.; ^3^ Graduate Program in Fundamental Nursing, Ribeirão Preto, Sao Paulo, Brazil.

**Keywords:** human papillomavirus, primary health care, Family Health Strategy, HPV vaccine

## Abstract

**Introduction:**

The coverage of the human papillomavirus (HPV) vaccine remains low worldwide. The Family Health Strategy (FHS) in Brazil has an important role in health promotion in communities. Given the FHS's close contact with assisted communities, the coverage of the HPV vaccine should be high in children. This study aims to investigate the acceptance of the HPV vaccine of parents or guardians of a peripheral community of the Brazilian Amazon region assisted by the FHS and influencing factors.

**Methods:**

A cross-sectional community-based study recruiting the residents of a subnormal agglomerate of Belém (Pará-Brazil) and covered by the FHS was conducted. Data were collected from September 30 to November 5, 2019. The questionnaire “Knowledge and Acceptability of HPV and Its Vaccine” was used. Data were analyzed through binary and multiple regression analyses.

**Results:**

A total of 247 participants were included in this study, and 85 of which (34.4%) declared that they did not vaccinate their children. Hesitation to vaccinate was related to few years of schooling (odds ratio [OR]: 0.79, *p*=0.008), having sons (OR: 3.14, *p*=0.000), inadequate knowledge about doses of the HPV (OR: 2.44, *p*=0.015), and knowledge of anyone who received the HPV vaccine (OR: 7.07, *p*=0.000).

**Conclusion:**

Results suggested the low efficiency of FHS in increasing the HPV vaccination coverage in the assisted communities. A strategy involving a dialog with assisted families and continuous health education to health professionals should be implemented to combat fake news and increase HPV vaccination coverage.

## Introduction

The human papillomavirus (HPV) causes different types of cancer: anal, vulvar, vaginal, oropharynx, and penile^1,2^ and is strongly associated with cervical cancer (CC).^3^ Of the estimated 570,000 CC cases, 84% occur in countries with a low human development index.^4,5^ Although the HPV vaccine has been available for more than a decade, the incidence of CC and other types of cancer caused by HPV remains high.^1,6^

In 2020, the World Health Organization (WHO) launched a strategy to eliminate CC in 2030. However, of the 194 member countries of the WHO, only 107 implement HPV vaccine programs and only 5 achieve the goal of 90% vaccination coverage.^6^ In accordance with the WHO,^5^ less than 25% of low-income countries have introduced HPV vaccine programs, and this value is extremely low compared with the 85% of high-income countries.

In Brazil, the National Immunization Program adopted the quadrivalent HPV vaccine only in 2014. Initially, only girls aged 11–13 years are included in the vaccination program. Currently, the HPV vaccine is available for girls aged 9–14 years, boys aged 11–14 years, and immunocompromised individuals. The vaccine is delivered predominantly through the Primary Health System at a two-dose schedule for free.^7,8^

The Primary Health System, which is integrated in the community and responsible for public vaccination, is an important tool for the massive vaccination of a population, but has failed to expand the HPV vaccination coverage. Brazilian studies obtained alarming data. Only 58.4% of females have completed the two-dose schedule, and 71.1% received only one dose in seven Brazilian cities, including Belém.^8^ In Western Amazon, 53.9% of females are not vaccinated.^9^ The midwest and north regions of Brazil have 14.57% and 9.24% vaccination coverage, respectively.^10^

Parents or guardians play important roles in vaccine hesitancy (VH) because their permission is needed for the vaccination of children. VH is defined as delaying vaccine acceptance or complete refusal even on conditions that information and vaccination services are available.^11,12^ In a specific territory, VH can vary among subpopulations due to influencing socioeconomic aspects. For example, VH can be high among people with low socioeconomic conditions and education, restricted access to information technologies, limited knowledge about effectiveness, risk and safety of the vaccine, disease that the vaccine covers, and risk for the disease.^12,13^

Worldwide, regarding HPV VH, many factors have been associated with low income and education,^14^ low knowledge about the HPV and the vaccine,^8,14,15^ absence of knowledge about other parents with children vaccinated against HPV and the antivaccine movement, and the belief that the HPV vaccine stimulates a precocious sexual life.^7,8,16,17^ The primary health care system, which is in close contact with communities, has an important role on decreasing the VH.

In Brazil, the Family Health Strategy (FHS) is the main entry point of people in the public health care system and is in close contact with the assisted population in the community where it is integrated. The FHS has three main attributes for health promotion: family orientation, community orientation, and cultural competence. Each FHS team is composed by a medical doctor, a nurse, a nursing technician, and community health agents (CHAs).^18^ Therefore, the FHS is expected to increase HPV vaccination coverage considerably in FHS territories. However, to date, no study has evaluated how FHS influences the parent's HPV vaccine acceptance. Therefore, this study aims to analyze the parents' HPV vaccine acceptance and its associated factors in a peripheral community of Belém covered by FHS.

Belém, the largest city in the Brazilian Amazon region, is the capital of Pará and has an estimated population of 1,506,420 and area of 1,059,466 square kilometers. Approximately 38.5% of the population lives in subnormal agglomerate characterized by an irregular urban pattern, lack of essential public services, and housing located in areas considered unsuitable for occupation.^19^ In 2020, Pará is the eighth Brazilian state to report a large number of CC cases (780). CC, the prevalent cancer type in women in the state, has incidence rates of 18.41 and 12.41 (100,000 inhabitants) in Belém.^20^

## Methods

### Study design and settings

This observational, cross-sectional, and community-based study was carried out in the Montese neighborhood of Belém. This area is a subnormal agglomerate, has an estimated population of 16,000, and has the largest number of FHS teams: FHS-I, FHS-II, FHS-III, and FHS-IV. Each FHS team assists 4000 inhabitants.^21^ The population of girls and boys within the age range of HPV vaccination in Montese is composed by 5556 children.^19^

In our study, we selected a microarea covered by the FHS-IV team because it has the highest number of CHAs (08). In this microarea, each team covered 756 houses. Data were collected from September 30 to November 5, 2019. Inclusion criteria were as follows: (1) parents or guardians, (2) aged 18 years or older, (3) living in a neighborhood, and (4) having children in the HPV vaccination age range. Parents or guardians unaware of their children's vaccination status were excluded.

The StatCalc (population survey) and Epi Info version 7.2.2.16 (Center for Disease Control and Prevention, Atlanta, Georgia, USA) were used to calculate the sample size on the basis of the 756 houses and an acceptable margin of error of 5%, 95% confidence interval (CI), design effect of 1.0, 20% expected frequency of unvaccinated persons against HPV,^22^ and 19% missing data.

After sample size calculation, we accessed the record of all families assisted by the FHS-IV team. All houses of families (379) with children in the age range of the HPV vaccine were visited. Finally, the number of such children in each house was entered into a digital spreadsheet, and the simple random probability sampling was carried out using a computer-generated list in the Biostat 5.3. When no one was at home on the day of the visit, the next house on the list was selected. A sample loss was the 34.82% (132 families/houses).

Data collection was conducted by trained researchers who understood the purpose of the study, form of data collection, and the need for confidentiality. These researchers were accompanied by CHA. Data were collected in the morning because of low rate of violence, and the average duration of data collection was 30 min.

During the domiciliary visit, the interview was limited to only one of the parents or guardians. When a house had more than one child, participants were asked to answer questions about the vaccination status of the oldest child (referred to as the “indicated” or “selected”).

### Data source and variables

The formulary used in this study was composed of two parts. The first part was used to characterize the sample of the study, whereas the second part was used to evaluate the parents' knowledge regarding the HPV vaccine and barriers and their acceptability of the vaccine. The sociodemographic formulary contained the following sociodemographic questions about children (i.e., age, years of schooling, sex, and vaccination status) and parents or guardians (i.e., degree of relatedness with children, sex, age, marital status, monthly family income, educational attainment, and number of children in the house).

To evaluate the HPV knowledge and the vaccine acceptance of the children's parents, we used the adapted version of the formulary “Knowledge and Acceptability of HPV and Its Vaccine.”^23^ This version contained 31 questions divided into six domains: (1) knowledge about HPV (7 questions), (2) knowledge about the HPV vaccine (11 questions), (3) vaccination barriers for HPV (2 questions), (4) acceptability of the HPV vaccine (3 questions), (5) personal background (3 questions), and (6) health professionals (4 questions). All questions had three options for answers: “no,” “yes,” and “I do not know.” In the adapted version of the formulary, we used only domains 1, 2, 3, and 4. Domains 5 and 6 were excluded because they were directed to health workers.

In addition, we revised question 16 of domain 2 from “Are 3 doses required for complete vaccination?” into “Are 2 doses required for complete vaccination?” due to the update on the number of vaccine doses. All modifications were consented previously by the author of the formulary.

Following the modifications, a pretest of the formulary was done in the study area with a random sample of 25 parents or guardians who were excluded from the study. After this pretest, no adjustment in the form or the content was necessary. During the formulary application, the interviewer cleared possible parents' doubts concerning the questions.

All participants were asked about their children's vaccination status. Responses were categorized into two groups: “vaccinated” (at least one dose) and “not vaccinated” (no dose). If the participant did not know the vaccination status, he was excluded. Vaccination status was the binary dependent variable. The response event selected was “not vaccinated.” Independent variables were sociodemographic factors (continuous variables: grade [year of schooling], age [in years], and number of children in the house), knowledge about HPV, knowledge about the HPV vaccine, barriers to HPV vaccines, and acceptability of the HPV vaccine. No recompense was offered to volunteers who participated in the study.

### Statistical analysis

Data were entered into the Epi Info, and analyses were performed using the Minitab 20 (Minitab, Inc., State College, PA) and BioEstat 5.3 (Instituto de Desenvolvimento Sustentável Mamirauá, Belém, Pará, Brazil) software. Categorical variables were expressed as percentages, and numerical variables were presented as means and standard deviations. Parameter estimation was used to describe the proportion of children that were not vaccinated against HPV. Multiple logistic regression was used in examining the factors associated with vaccination status.

Binary regression analysis was used in assessing the association of the independent variables with the dependent variable. All variables with *p*-values <0.20 were entered into the multiple logistic regression. Multicollinearity was verified using the variance inflation factor (VIF), and correlations were tolerable at VIF <5. Crude and adjusted odds ratios (ORs) and their respective 95% CIs were used in evaluating the effects. *p*-Values <0.05 were considered statistically significant. We did not use any hierarchical model, and all tests used were two-sided.

### Ethical aspects

This study was approved by the Research Ethics Committee of the Federal University of Pará under protocol no. 3968426 and followed the principles of the Declaration of the Helsinki. All participants provided written informed consent to participate in the study. None of the participants received rewards for participating in the study.

## Results

In this study, we visited 379 houses and the sample of this study was composed by 247 parents or legal guardians (only one legal guardian refused to participate in the study) having in average of 2.8 children per household in the age range of the HPV vaccine. Although, the sample calculus resulted that we had to interview a number of family covering at least 360 children in specific age range for HPV vaccine, the number of houses visited covered about 692 children.

The children's parents or guardians had a mean age of 41.4±12.1 years (range: 18–79 years). Most of the participants were females (89.5%, 221/247), had a monthly family income of less than two minimum wages (90.6%, 203/224), were mothers (62.3%, 154/247), and had high school or university education (60.7%, 150/247). Only half of the participants were unmarried (single/separated/divorced/windowed, 51.4%, 127/247). The average number of children in the house was 2.8, and 53.8% of the children (133/247) were girls with a mean age of 12 years. Approximately 98.4% attended school (243/247; Table 1).

**Table 1.** Sociodemographic Factors Associated with Human Papillomavirus Vaccination in Brazilian Amazon

Variable sociodemographic	Total	Vaccinated against HPV		Binary regression		
		No, ***n*** (%)	Yes, ***n*** (%)	OR	(95% CI)	***p***
Participant or guardian						
Mother	154 (62.3)	51 (33.1)	103 (66.9)		Ref.	
Grandmother or grandfather	45 (18.2)	15 (33.3)	30 (66.7)	1.01	(0.49–2.04)	0.98
Father	20 (8.1)	8 (40.0)	12 (60.0)	1.35	(0.51–3.50)	0.54
Uncle or aunt	17 (6.9)	6 (35.3)	11 (64.7)	1.10	(0.38–3.14)	0.86
Others	11 (4.5)	5 (45.5)	6 (54.5)	1.68	(0.49–5.77)	0.41
Sex						
Female	221 (89.5)	74 (33.5)	147 (66.5)		Ref.	
Male	26 (10.5)	11 (42.3)	15 (57.7)	0.69	(0.30–1.56)	0.37
Marital status						
Married/stable union	120 (48.6)	38 (31.7)	82 (68.3)		Ref.	
Single/separated/divorced/windowed	127 (51.4)	47 (37.0)	80 (63.0)	1.27	(0.74–2.14)	0.38
Family income (minimum wage)^a^						
<2 minimum wages	203 (90.6)	73 (36.0)	130 (64.0)	2.39	(0.77–7.36)	0.13
>2 minimum wages	21 (9.3)	4 (19.0)	17 (81.0)		Ref.	
Do not know^b^	23	8	15			
Schooling						
Illiterate/elementary	97 (39.3)	30 (30.9)	67 (69.1)	0.77	(0.44–1.33)	0.35
High school/university	150 (60.7)	55 (36.7)	95 (63.3)		Ref.	
Age, in years						
Mean (SD)	41.4	40.8	41.8	1.01	(0.98–1.02)	0.61
Number of children in the household						
Mean (SD)	2.8 (1.6)	2.8	2.7	0.97	(0.82–1.13)	0.72
Age, in years						
Mean (SD)	12 (1.4)	11.8	12.1	0.85	(0.71–1.02)	0.09
Year of schooling						
Mean (SD)	6 (1.7)	5.6	6.1	0.82	(0.70–0.96)	0.01
Sex						
Female	133 (53.8)	34 (25.6)	99 (74.4)		Ref.	
Male	114 (46.2)	51 (44.7)	63 (55.3)	2.36	(1.37–4.03)	0.00
Attending school						
No	4 (1.6)	2 (50.0)	2 (50.0)	1.93	(0.26–13.9)	0.52
Yes	243 (98.4)	83 (34.2)	160 (65.8)		Ref.	

Pará, Brazil, 2019.

^a^ Brazilian minimum wage in November 2019—R$ 998 real per month (=U$237.61).

^b^ Not considered for statistical calculation.

CI, confidence interval; HPV, human papillomavirus; OR, odds ratio; Ref., reference category; SD, standard deviation.

The proportion of participants who reported that their children did not receive the HPV vaccine was 34.4% (85/247, 95% CI: 28.5–40.3%). Participants with male children were twice as likely to not vaccinate their children compared with participants with female children (OR: 2.36, 95% CI: 1.37–4.03, *p*=0.00). The probability of having an unvaccinated status decreased with increasing number of years of schooling of children (OR: 0.86, CI: 0.78–0.95, *p*=0.003; Table 1).

Regarding knowledge about HPV vaccination, participants who answered “no” or “not sure” to questions about the need for two doses for a complete vaccination were twice as likely to not vaccinate their children (OR: 2.16, 95% CI: 1.14–4.07, *p*=0.02; Table 2).

**Table 2.** Knowledge About HPV and HPV Vaccine Associated with Human Papillomavirus and Associated with Human Papillomavirus Vaccination in Brazilian Amazon

Knowledge about HPV	Total	Vaccinated against HPV		Binary regression		
		No, ***n*** (%)	Yes, ***n*** (%)	OR	(95% CI)	***p***
Do you know what HPV is?						
No/not sure	148 (59.9)	57 (38.5)	91 (61.5)	1.59	(0.91–2.74)	0.10
Yes	99 (40.1)	28 (28.3)	71 (71.7)		Ref.	
Is HPV a virus?						
No/not sure	75 (30.4)	23 (30.7)	52 (69.3)	0.78	(0.43–1.40)	0.41
Yes	172 (69.6)	62 (36.0)	110 (64.0)		Ref.	
Is HPV a sexually transmitted disease?						
No/not sure	83 (33.6)	29 (34.9)	54 (65.1)	1.04	(0.59–1.80)	0.90
Yes	164 (66.4)	56 (34.1)	108 (65.9)		Ref.	
Can HPV cause cervical cancer?						
No/not sure	52 (21.0)	20 (38.5)	32 (61.5)	1.25	(0.66–2.35)	0.49
Yes	195 (79.0)	65 (33.3)	130 (66.7)		Ref.	
Can HPV cause changes in the Pap (screening for cervical cancer)?						
No/not sure	102 (41.3)	36 (35.3)	66 (64.7)	1.07	(0.62–1.81)	0.81
Yes	145 (58.7)	49 (33.8)	96 (66.2)		Ref.	
Is cervical cancer a major cause of cancer in women?						
No/not sure	26 (10.5)	11 (42.3)	15 (57.7)	1.46	(0.63–3.32)	0.37
Yes	221 (89.5)	74 (33.5)	147 (66.5)		Ref.	
Can smoking increase the risk of cervical cancer?						
No/not sure	101 (40.9)	33 (32.7)	68 (67.3)	0.88	(0.51–1.49)	0.63
Yes	146 (59.1)	52 (35.6)	94 (64.4)		Ref.	
Does the HPV vaccine prevent cervical cancer?						
No/not sure	47 (19.0)	18 (38.3)	29 (61.7)	1.23	(0.63–2.37)	0.53
Yes	200 (81.0)	67 (33.5)	133 (66.5)		Ref.	
Should the HPV vaccine be given before the first sexual intercourse?						
No/not sure	70 (28.3)	29 (41.4)	41 (58.6)	1.53	(0.86–2.70)	0.15
Yes	177 (71.7)	56 (31.6)	121 (67.4)		Ref.	
Can the HPV vaccine be given to people who have had sex?						
No/not sure	109 (44.1)	36 (33.0)	73 (67.0)	0.90	(0.52–1.52)	0.68
Yes	138 (55.9)	49 (35.5)	89 (64.5)		Ref.	
Can the HPV vaccine be harmful to health?						
No	58 (23.5)	24 (41.4)	34 (58.6)	1.48	(0.80–2.71)	0.20
Yes/not sure	189 (76.5)	61 (32.3)	128 (67.7)		Ref.	
Can the HPV vaccine cause HPV infection?						
No	90 (36.4)	31 (34.4)	59 (65.6)		Ref.	
Yes/not sure	157 (63.6)	54 (34.4)	103 (65.6)	1.00	(0.58–1.72)	0.99
Is the HPV vaccine provided by the government?						
No/not sure	16 (6.5)	8 (50.0)	8 (50.0)	2	(0.72–5.53)	0.18
Yes	231 (93.5)	77 (33.3)	154 (66.7)		Ref.	
Is the HPV vaccine part of the girls' immunization record?						
No/not sure	43 (17.4)	17 (39.5)	26 (60.5)	1.31	(0.66–2.57)	0.44
Yes	204 (82.6)	68 (33.3)	136 (66.7)		Ref.	
Where did you hear about the HPV vaccine?						
TV, radio, internet	145 (58.7)	46 (31.7)	99 (68.3)	0.85	(0.48–1.50)	0.57
Others	17 (6.9)	9 (52.9)	8 (47.1)	2.06	(0.72–5.90)	0.17
Health professional/school	85 (34.4)	30 (35.3)	55 (64.7)		Ref.	
Are two doses required for complete vaccination?						
No/not sure	49 (19.8)	24 (49.0)	25 (51.0)	2.16	(1.14–4.07)	0.02
Yes	198 (80.2)	61 (30.8)	137 (69.2)		Ref.	
Does the HPV vaccine lessen the chance of having genital warts?						
No/not sure	112 (45.3)	38 (33.9)	74 (66.1)	0.96	(0.56–1.62)	0.88
Yes	135 (54.7)	47 (34.8)	88 (65.2)		Ref.	
Does the HPV vaccine decrease the chance of having Pap (cervical cancer screening) changes?						
No/not sure	97 (39.3)	32 (33.0)	65 (67.0)	0.90	(0.52–1.54)	0.71
Yes	150 (60.7)	53 (35.3)	97 (64.7)		Ref.	

Pará, Brazil, 2019.

No statistically significant association was found between variables on “HPV Vaccine Barriers” and acceptance of HPV vaccine. However, on the acceptability of the HPV vaccine, participants who answered “no” or “not sure” to the question “Do you know anyone who already had the HPV vaccine?” were six times likely to have unvaccinated children (OR: 6.47, 95% CI: 3.09–13.5, *p*=0.00; Table 3).

**Table 3.** Barriers and Acceptability to Human Papillomavirus Vaccine Associated with Human Papillomavirus Vaccination in Brazilian Amazon

Barriers and acceptability of HPV vaccine	Total	Vaccinated against HPV		Binary regression		
		No, ***n*** (%)	Yes, ***n*** (%)	OR	(95% CI)	***p***
HPV vaccine barriers						
Do you think the HPV vaccine would stimulate the onset of sexual life earlier?						
No	180 (72.9)	61 (33.9)	119 (66.1)		Ref.	
Yes/not sure	67 (27.1)	24 (35.8)	43 (64.2)	1.09	(0.60–1.95)	0.78
Do you think that after the HPV vaccine still the necessity of the condom usage?						
No/not sure	9 (3.6)	3 (33.3)	6 (66.7)	0.95	(0.23–3.90)	0.95
Yes	238 (96.4)	82 (34.5)	156 (65.5)		Ref.	
Do you think that after the HPV vaccine you still need to have the Pap test (cervical cancer screening)?						
No/not sure	8 (3.2)	2 (25.0)	6 (75.0)	0.63	(0.12–3.17)	0.57
Yes	239 (96.8)	83 (34.7)	156 (65.3)		Ref.	
Acceptability of HPV vaccine						
Do you know anyone who has already had the HPV vaccine?						
No/not sure	41 (16.6)	29 (73.7)	12 (29.3)	6.47	(3.09–13.5)	0.00
Yes	206 (83.4)	56 (27.2)	150 (72.8)		Ref.	
Have you taken the HPV vaccine yet?						
No	199 (80.6)	68 (34.2)	131 (65.8)		Ref.	
Yes/not sure	48 (19.4)	17 (35.4)	31 (64.6)	1.1	(0.54–2.04)	0.87
Would you recommend the HPV vaccine to a child, friend, or relative?						
No/not sure	7 (2.8)	1 (14.3)	6 (85.7)	0.31	(0.03–2.61)	0.28
Yes	240 (97.2)	84 (35.0)	156 (65.0)		Ref.	

Pará, Brazil, 2019.

All variables with *p*-values ≤0.2 in the bivariate analysis in all domains (i.e., sociodemographic factors, knowledge about HPV vaccine, and acceptability of HPV vaccine) were then explored through multiple logistic regression analysis (Table 4).

**Table 4.** Results of the Multiple Logistic Regression Analysis

Variables	Adjusted OR	(95% CI)	***p***	VIF
Sociodemographic				
Year of schooling of children (as a continuous measure)				
	0.79	(0.66–0.94)	0.008	1.02
Sex—daughter or son—(daughter as reference)				
Male	3.14	(1.70–5.79)	0.000	1.05
Knowledge about HPV vaccine				
Are two doses required for complete vaccination? (Yes as reference)				
No/not sure	2.44	(1.19–4.99)	0.015	1.02
Acceptability of HPV vaccine				
Do you know anyone who has already had the HPV vaccine? (Yes as reference)				
No/not sure	7.07	(3.11–16.0)	0.000	1.02

Pará, Brazil, 2019.

VIF, variance inflation factor.

The likelihood of being unvaccinated decreased with increasing years of schooling of children (OR: 0.79, CI: 0.66–0.94, *p*=0.008). Male children were three times likely to be unvaccinated than female children (OR: 3.14, 95% CI: 1.70–5.79, *p*=0.000). Participants with inadequate knowledge about the doses of the HPV vaccine were twice more likely to have unvaccinated children than those with adequate knowledge (OR: 2.44, 95% CI: 1.19–4.99, *p*=0.015). Participants who answered “no” or “not sure” to the question on whether they know anyone who already had the HPV vaccine were seven times likely to refuse the vaccination of their children (OR: 7.07, 95% CI: 3.11–16.0, *p*=0.000).

## Discussion

Our study showed HPV vaccination coverage of 65.6% of in the study area covered by the FHS. Approximately 34.6% of the children's parent or guardians did not accept the vaccine, which was associated with having a son, not knowing the number of necessary vaccine doses, and not knowing anyone vaccinated. The years of schooling of children were directly associated with the acceptance of the HPV vaccine by parents or guardians.

This study had some limitations. First, the coverage of the HPV vaccine was based on parental report because records on health information systems about HPV community vaccination coverage were unavailable. Second, recall bias and social desirability cannot be avoided. To limit these biases, we requested the children's vaccination records. Third, our study design and sample methods were limited to only one area covered by one FHS team and therefore the results of this study cannot be extrapolated for the Brazilian population or other areas covered by FHS. However, our results bring an alert to health authorities to implement improvement in FHS network. To see the real impact of the FSH in the HPV vaccine in Brazil, new studies should be done comparing the results among the Brazilian regions.

The HPV vaccination coverage in the Montese (65.6%) was higher than that in Belém (58.4%), which was possibly because of the influence of the FHS in this area. However, the HPV vaccination coverage in Montese remains far from the target goal of 90% proposed in the global strategy for eliminating CC. Considering that FHS is in close contact with people living in the assisted communities, we expect a high HPV vaccination coverage. However, our results showed that the HPV vaccination in this neighborhood was quite close to that in Brazil. A previous study showed HPV vaccination values of 75.6%, 79.7%, and 65.9% in children aged 13, 14, and 15 years, respectively.^24^ These factors indicate the necessity of coverage expansion and investments on FHS in Belém and Brazil. Only 303,600 inhabitants of Belém (20.4%) are covered by FHS services.^19^

About 34.6% of our sample did not accept the HPV vaccine. This problem is not restricted to Brazil. In Uganda, of the 6093 girls who participated in a study, only 22% are vaccinated against HPV.^25^ In Germany, this percentage is even smaller (17.4% of 4747 vaccinated girls).^26^ However, through being in close contact with the families, the FHS team has the preponderant role of demystifying myths, earning the trust of the assisted population, and promoting an increase in the vaccination coverage in the community. In Georgia, United States of America, a study showed that the caregivers' inability of earning the trust of the children's parents is the reason to HPV VH.^27^

Parents or guardians with male children had a high rate of nonacceptance of the HPV vaccine (44.7%) because of their insufficient knowledge about the effect of HPV on men's health. In Sweden and United States, the low level of vaccination in boys is associated with their parents' absence of knowledge about the importance of vaccination to boys and their belief that their sons are not at risk of acquiring the HPV.^28,29^ Another study involving Latin American mothers from Colombia, Guatemala, El Salvador, Dominican Republic, United States, and Puerto also showed a low acceptability of the HPV vaccine for boys.^30^

The societal historical construction restricts HPV as a problem limited to women, and this belief needs to be demystified. Progress has been made toward the HPV vaccine among boys but remains lacking. Globally, 107 countries introduced the HPV vaccine, but only 33 countries, including Brazil, reported a gender-neutral HPV vaccination program in their vaccination calendars. In these countries, the HPV vaccination coverage for males often rapidly converges with that for females.^6^ These facts show the necessity to reinforce to the society the benefits of vaccination for boys to prevent the several types of cancer in men promoted by HPV.

The likelihood of not being vaccinated decreases with increasing years of schooling. This finding suggests the success of the school health program launched in 2007 by the Brazilian Ministry of Health. The program's primary goal is health promotion in students, including reproductive health. This program is associated with the FHS, and one of the functions of professional workers is to keep vaccination cards updated.^31^ In addition, given their knowledge about HPV and vaccine benefits, students are likely to accept the vaccine and convince their parents. A previous study on Chinese university students showed that a high educational attainment results in low HPV vaccine hesitation.^32^

In this study, parents or guardians who did not have their children vaccinated were likely to have no knowledge on the number of doses needed. This fact could be related to the lack of knowledge about the HPV vaccine. In a district of Utah, low HPV vaccination coverage is directly associated with parents lacking knowledge about the benefits of HPV vaccine.^33^ In Italy, the parents' HPV VH is directly associated with lack of knowledge about HPV, vaccine, and usefulness of the vaccine for HPV-related cancer prevention.^34^

In the multiple regression analysis, participants who answered “no” or “not sure” to questions about knowing anyone who already got the HPV vaccine were likely to have vaccinated children. Given that the HPV vaccine programs have been launched worldwide, HPV antivaccine campaigns are spreading fake news and acting as barriers to the acceptance of the vaccine. In the United States, the followers of an antivaccine group in Facebook has increased to ∼298% since 2019 with more than 31,000,000 followers.^35^

In 2017, a study on 1263 parents in the United States showed 19% refusal to vaccinate their children after reading stories about the vaccine's harmful effects.^36^ Knowledge of people who are vaccinated and have not shown side effects increases the acceptance of the vaccine. A previous study in the Brazilian Amazon region showed that adolescents who know someone vaccinated are twice as likely to be vaccinated.^9^

Being in close contact with the community, the FHS needs to strategize a dialog with families to reduce myths and promote health in communities. However, although health professionals have a preponderant role on the HPV HV elimination, a study in Italy showed that pediatricians working in Primary Healthcare System do not have expected conduct regarding the HPV vaccine prescriptions. For example, some of pediatricians revealed not prescribing the vaccine for boys for considering it unsafe or unnecessary to them,^37^ suggesting the need for health authorities to provide health updating and standardize the process of professionals.

FHS should also promote the reduction of HPV HV through social media. For example, the *HPV Vaccination NOW* campaign on social media has been successful in correcting misinformation about the HPV vaccine by promoting peer-to-peer dialog.^35^ Most of the neighborhood of Belém has pages on Facebook and Instagram. In Germany, a social media campaign was launched in 2017 to increase the HPV vaccine acceptance. From 2017 to 2018, the positive comments on the social media page increased from 50% to 75%, and the page reached more than 8,020,000 followers in 2019.^38^

## Conclusion

Although our results showed that the HPV vaccination coverage in an FHS-covered community is higher than that in Belém, parents still refuse the vaccine. Nonacceptance is directly associated with limited knowledge about the HPV and its vaccine. Being integrated into communities and in close and constant contact with families, the FSH needs to hold dialogs with families to combat antivaccine campaigns. The use of social media should be considered as a strategy. Furthermore, continuous health education should be ensured to enable CHAs to deal with this subject with families.

Community educational interventions and health workers' continuing education highlighting the importance and efficiency of the HPV vaccine should be carried out to increase HPV vaccine acceptance. Brazilian Municipal, State, and Federal health authorities should provide resources and infrastructure to FHS for improved performance.

## Abbreviations Used

CC: cervical cancer
CHAs: community health agents
CI: confidence interval
FHS: Family Health Strategy
HPV: human papillomavirus
OR: odds ratio
SD: standard deviation
VH: vaccine hesitancy
VIF: variance inflation factor
WHO: World Health Organization
